# Development of an Extended Reality Simulator for Basic Life Support Training

**DOI:** 10.1109/JTEHM.2022.3152365

**Published:** 2022-02-16

**Authors:** Dong Keon Lee, Haneul Choi, Sanghoon Jheon, You Hwan Jo, Chang Woo Im, Song Young Il

**Affiliations:** Department of Emergency MedicineSeoul National University Bundang Hospital 65462 Seongnam 13620 Republic of Korea; Department of Emergency MedicineSeoul National University College of Medicine 37990 Seoul 03080 Republic of Korea; TETRASIGNUM Corporation Seoul 05839 Republic of Korea; Department of Thoracic and Cardiovascular SurgerySeoul National University Bundang Hospital, Seoul National University College of Medicine 65462 Seoul 03080 Republic of Korea; THIRTEENTH FLOOR Corporation Seoul 06798 Republic of Korea

**Keywords:** Basic life support, cardiopulmonary resuscitation, extended reality, BLS training, virtual reality

## Abstract

Objective: Extended Reality (XR) is a simultaneous combination of the virtual and real world. This paper presents the details of the framework and development methods for an XR basic life support (XR-BLS) simulator, as well as the results of an expert usability survey. Methods: The XR-BLS simulator was created by employing a half-torso manikin in a virtual reality environment and using BLS education data that is in line with the 2020 American Heart Association guidelines. A head-mounted display (HMD) and hand-tracking device were used to perform chest compressions and ventilation and to enable the use of an automated external defibrillator in a virtual environment. A usability study of the XR-BLS simulator through an expert survey was also conducted. The survey consisted of a total of 8 items: 3, 2, and 2 questions about the ease of use of XR-BLS, delivery of training, and artificial intelligence (AI) instructor in the simulator, respectively. Results: The XR simulator was developed, and the expert survey showed that it was easy to use, the BLS training was well delivered, and the interaction with the AI instructor was clear and understandable. Discussion/Conclusion: The XR-BLS simulator is useful as it can conduct BLS education without requiring instructors and trainees to gather.

## I. Introduction

In Europe and the United States, over 300,000 out-of-hospital cardiac arrests (OHCAs) occur annually [1], [2]. The prognosis of OHCA is so poor that only a few patients show a good neurological outcome that enables them to return to daily life. To improve the outcomes of OHCA patients, there have been many efforts including basic life support (BLS) training to increase the rate of early recognition and bystander cardiopulmonary resuscitation (CPR). However, even with those efforts, the rate of bystander CPR is still below 50% [3], [4].

Prevailing BLS training sessions rely on the use of a manikin, a training video and, more recently, a feedback device. Although the quality of education improves after the addition of the feedback device, conventional BLS training conducted with manikins in a simulation room is still neither realistic nor immersive. Conventionally, trainees and instructors are required to gather in the same place and at the same time to conduct the training. These spatial and temporal constraints are not only inconvenient but also must not be present in modern society in which many technologies are developed. In particular, since the start of the COVID-19 pandemic, social distancing and noncontact policies around the world have been leading the way in preventing the spread of the pandemic, and it has become difficult for trainees to gather and listen to an instructor’s lecture and learn the procedure under their guidance.

Virtual reality (VR), which refers to devices that keep users from seeing the real world and instead allow them to view digitally rendered images, has recently been developed and applied to cardiopulmonary resuscitation (CPR) training [5]. VR may offer procedures such as chest compression, ventilation, and defibrillation in a realistic environment, but they cannot provide real-life sensations [6].

A view of the physical world that is enhanced by digital information is termed augmented reality (AR). For a spectrum of VR and AR that combines the real world with the digital world, the term mixed reality (MR) is used. MR, however, is sometimes used differently depending on its precise definition. This is why “extended reality” (XR) has been proposed as an umbrella term encompassing AR, VR, and MR.

XR, a simultaneous combination of the virtual and the real world, can be used to deliver CPR training in immersive and realistic environments with a similar level of instruction to conventional CPR training. Furthermore, trainees and instructors are not required to be gathered during COVID-19 outbreaks, and trainees are able to complete the training without human supervision.

Based on the above advantages, we recently developed an XR-based basic life support (BLS) simulator. This paper describes the development of an XR-BLS simulator and how it works, including the technologies and a concept of an immersive and realistic XR-based training system. In addition, the results of an expert survey, which was conducted with 16 experts from 6 different institutions, are discussed.

## II. Methods and Procedures

### A. Development of XR-Based BLS Simulator

#### 1) Manikin Recognition in VR

As manikins are not produced as trackable objects in VR, it is necessary that the manikin in the VR environment match the real manikin in front of the user perfectly. Initially, the manikin was set in place and tracked in the VR environment using the Vive Tracker (HTC Corporation, Taoyuan, Taiwan). However, because of discrepancies in the data exchange between the tracker and the base station, another method was devised to manually calibrate the manikin by moving it within the VR world with the Vive Controller (HTC Corporation, Taoyuan, Taiwan). Therefore, a calibration mode was added to the program where the user was able to see the manikin and to adjust its position by holding down the trigger button on the Vive Controller. The manikin can be moved along the X, Y, and Z-axis and can be rotated around the Y-axis for more accurate calibration.

#### 2) Chest Compressions & Rescue Breaths in VR

The ability to perform chest compressions accurately and give rescue breaths quickly while wearing the headset meant that the real manikin had to align perfectly with the VR manikin.

Initial tests indicated that learners had difficulty locating various parts of the manikin even after calibration, which led to the development of a short tutorial to help them become familiar with the VR environment. This process was intended to make it easier for learners who might not have much experience with the VR to find the correct location for chest compressions and rescue breaths.

During conventional CPR training, learners are taught to perform rescue breaths on the manikin by pinching its nose, covering the whole opening of the mouth. However, early trials during the research and development phase found that the learners could not physically reach the manikin’s mouth correctly and cover it completely while wearing a headset. This was because of the protrusion of the headset that blocked the learner’s face from getting close enough to perform rescue breaths on the manikin. To solve this problem, a separate mouthpiece was 3D-printed with Acrylonitrile Butadiene Styrene which was slotted on the manikin’s mouth to facilitate rescue breaths.

#### 3) Automated External Defibrillator (AED)

The use of AED in a VR environment is the same as in reality, however, it is only implemented in a VR environment, unlike chest compression and ventilation. In a VR environment, after powering on the AED, attach two pads to the patient’s chest and connect the pads connector to the AED. The electrocardiogram is then analyzed, and defibrillation is performed if necessary. It was implemented by touching the pad with a hand and then by touching it again on a specific part of the patient’s chest wall to pick up or attach the pad. Also implemented were power and defibrillation buttons that could be touched by hand.

#### 4) Data Collection

To collect as much data as possible regarding the learner’s performance, it was essential to keep track of each step of CPR training. This was made possible using various sensors and trackers. The sensors in the manikin track the learner’s actions for each step such as checking for a response, performing chest compressions, and performing rescue breaths.

However, various other data had to be collected including the learner’s head position, hand position, and audio input for the steps checking for a response (calling out to the patient), calling for help (looking directly at a bystander, pointing at them and calling out for help), checking for breathing (looking at the patient’s head, chest, and stomach to see if they are breathing), chest compressions (counting the number of chest compressions out loud) and using an AED (turning on the AED, placing the pads, administering the electric shock, asking the bystander to continue or stop chest compressions). Various methods have been developed to obtain these data. For example, head position data was tracked by using the Base Station 2.0 (HTC Corporation, Taoyuan, Taiwan) which synchronizes with the VR headset to locate its position. Hand tracking and position data were obtained through a Leap Motion sensor (Ultraleap, San Francisco, USA). Finally, audio input was tracked by using Unity’s native audio plugin (Unity Technologies, Copenhagen, Denmark).

#### 5) Data Management

A database of results was required to log and manage the incoming data. After the “practice” and “test” modes, learners were able to see their results on the kiosk screen and through the web portal. The data was also available to export to a Microsoft Excel spreadsheet file (Microsoft, Redmond, USA). The results saved in the database include checking for a response, calling for help, checking for breathing, number of chest compressions, mean compression depth, number of adequate compression depths, mean compression rate, adequate compression depth and rate, number of compressions with correct hand position, correct hand position, number of chest compressions with full release (n), compression and full release, hands-off time, mean ventilation volume, and AED use.

#### 6) Framework

The training application for the use of the XR-BLS simulator was developed according to the 2020 American Heart Association (AHA) guidelines for BLS [7]. The curriculum provided by the application follows three different modes, each specifically designed for CPR learning, practicing, and test purposes.

The learning mode consists of two parts – an introductory video explaining the steps for CPR, and an empty white room in which the steps are explained in much more detail by a VR instructor. The explanations are supplemented by 3D graphics, animations, and videos. Learners can perform the step immediately afterward on the manikin in front of them and that can be seen in the VR environment. The practice and test modes are set in a street environment. The practice mode has an instructor nearby to prompt the learner to perform each step of CPR, while the test mode involves a minimal intervention, which moves on to the next scene if the correct action is not taken after an allotted period, to test users on their CPR skills.

#### 7) System Design

**FIGURE 1.**
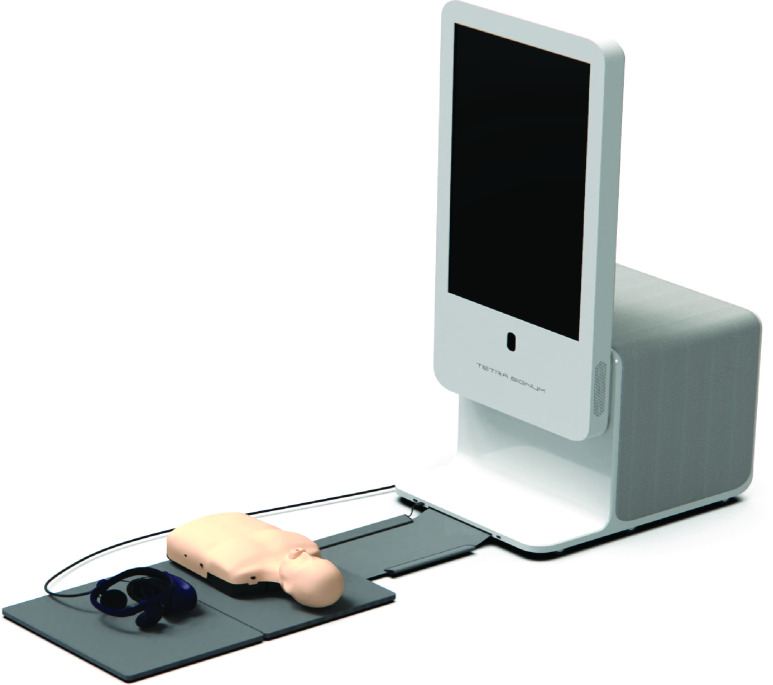
Extended reality basic life support (XR-BLS) Training Solution Setup. An image of the whole XR-BLS Training Solution setup, including the housing unit, touch screen, mat, manikin, and virtual reality headset.

The platform consists of the housing unit with an attachable base station, a touch screen for students to use to log in, play the content and check their results, a half-torso manikin (bestCPR, Gimpo, South Korea), a custom-made mat on which learners train, an HTC Vive Pro HMD (Head-Mounted Display) with an attached hand-tracking device (Leap Motion, UltraLeap, California, USA), the base station which is a device that tracks the location of the headset for head tracking, Vive Controller, and a computer to run the application (Fig. 1).

The next level of the system is the primary engine that was used to develop the application. The engine used was Unity coded in C# and included AVProVideo (Renderheads, Cape Town, South Africa) for video rendering, Obi (Virtual Method Studio, Madrid, Spain) for textures (primarily used in the AED model), SocketIO (Automattic, California, USA) for socket connectivity, and SteamVR (Valve Corporation, Washington, USA) to operate the VR device.

The database that held the learner’s information and details of their results for the practice and test modules was developed using Node.js and MySQL (Oracle, Texas, USA), with the platform itself being hosted on AWS (Amazon, Seattle, USA).

### B. User Experience (UX)

#### 1) HTC Vive Pro

The primary input for learners was via the use of their own hands, head, and voice.

Hand-tracking technology (Leap Motion tracker) allowed learners to be able to see 3D graphics of their real hands in action and to manipulate 3D objects (i.e., the AED).

The audio input was processed based on the sound intensity (decibels) only. Although the audio input did not encompass speech recognition, it provided a suitable experience for learners to interact with the 3D characters and to practice the relevant steps of CPR (e.g., checking for a response, calling for help, and using an AED) without requiring a specific script to complete them. The reason for simply measuring the sound intensity without recognizing specific words is that the simulator was intended for use in any country, regardless of its language. However, it is not easy to upgrade the program in order for it to can recognize speech in different languages.

Finally, to track where the learner was looking, head-tracking technology was implemented using the Base Station 2.0, also known as a ’lighthouse’, which can detect the location of the HMD.

#### 2) Manikin

**FIGURE 2.**
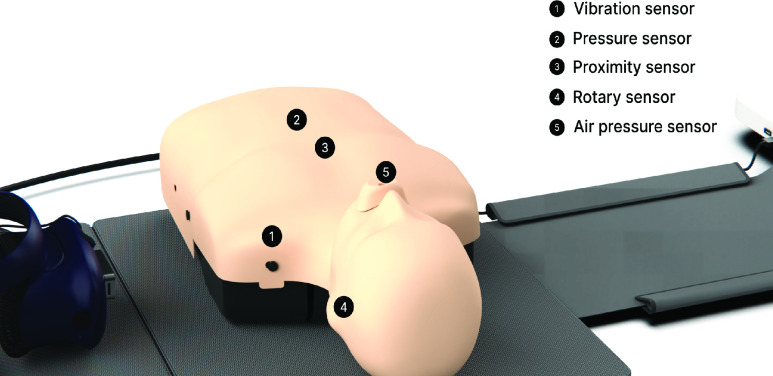
Manikin and sensors. The manikin and the embedded sensors: vibration, pressure, proximity, rotary, and air pressure sensors.

The manikin (Nurugo B100, BEST CPR Inc., Gimpo, South Korea) was equipped with five sensors as shown in Fig. 2. These sensors included a vibration detector (HX711, Avia semiconductor, Xiamen, China) to track the learner checking response, a load cell (LCB03, A&D Co. Ltd, Tokyo, Japan) to detect the depth and rate of the chest compression, a capacitance level sensor (MLDU01A, AD semiconductor, Seoul, South Korea) to detect the chest compression location, a rotary encoder (Arduino Encoder Sensor, EDUINO, Jeonju, South Korea) to detect the opening of the airway, and an air pressure sensor (33A series, SHIBA KOREA Co. Ltd., Anyang, South Korea) to determine the volume of the rescue breath. This raw data output by the sensors embedded in the manikin was converted into useful data through a circuit board and fed into the computer, producing a string of letters and numbers.

The chest compression depth and the air volume of the rescue breaths were estimated by equating the full sensor output ranges (minimum to maximum voltage outputs) to scales of 0–7 cm and 0–700 ml, respectively. For example, when the learner presses down on the chest, increasing the output by approximately 78%, this would show a compression depth of around 5.5 cm. If the trainee does not fully release the pressure after performing a chest compression and then presses again, the load cell connected to the chest spring of the manikin measures the value of the compression depth and informs of the incomplete relaxation.

In terms of ventilation, air only enters the lung bag when the manikin’s head is tilted. Therefore, when trainees perform ventilation, the rotary encoder sensor first detects the tilt of their heads, and then the air pressure sensor calculates how much air has entered the lung bag. Since the chest wall rise in the VR environment is not perfectly matched to that of an actual manikin, an air pressure sensor determines whether adequate ventilation has occurred.

#### 3) Environmental Assets and Characters

The 3D environment was primarily made up of the background and character. The ’bystander’ characters and the ’patient’ character were created using a variety of purchased assets which were then re-modeled and rigged using 3DS Max (Autodesk, San Rafael, USA). The ’bystander’ characters consisted of one female and one male character who, in the scenario, respectively call emergency services and go to fetch an AED.

The street environment was a combination of purchased assets that were also re-modeled as well as processed via lighting and texture baking. Other visual elements included 3D graphics in addition to videos and interactive objects (the AED) which aided in the learner’s initial CPR training.

The ’instructor’ character, named Aiden, was based on a real person and was created by 3D scanning, followed by rigging, and then animated using motion capture supplemented by animated movements and gestures.

**FIGURE 3.**
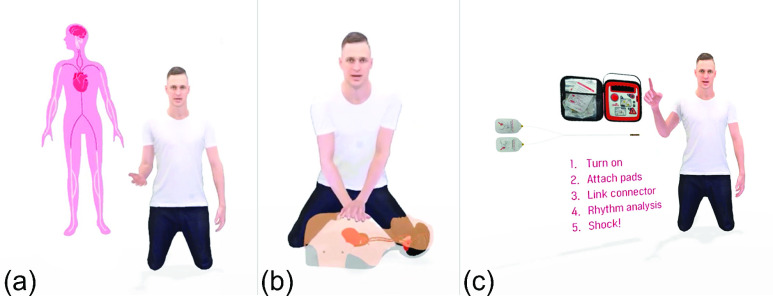
Examples of 3D Graphics used to supplement the ’Basic Learning’ course (a) 3D graphics of blood circulating through the body (b) blood reaching the brain (as shown within the manikin) (c) an automated electrical defibrillator and pads.

The instructor was programmed to provide real-time feedback based on the learner’s performance. For example, during chest compressions, the instructor gave feedback on whether the learner was counting aloud and also on the rate, depth, and location of the chest compressions. Other types of feedback included whether the learner spoke at a sufficiently loud volume (e.g., when asking the bystanders for help) or if they did not focus on the patient (e.g., when checking the patient’s consciousness). The instructor’s feedback comprised only simple assessments of the learner’s actions, such as incorrect position, fast/slow, strong/weak, as opposed to the more detailed information displayed in the graphical user interface (GUI), which included the chest compression depth, rate, and ventilation volume (Fig. 3).

#### 4) Audio

The learner’s experience was also complemented by audio assets that assisted with immersion, such as background ambiance and sound effects for an ambulance siren or the AED shock sound. Sound effects were also used as cues to indicate the learner’s progress, for example, if they were running out of time for each specific step or when they had completed the step correctly.

### C. User Interface (UI)

#### 1) Graphical User Interface (GUI)

**FIGURE 4.**
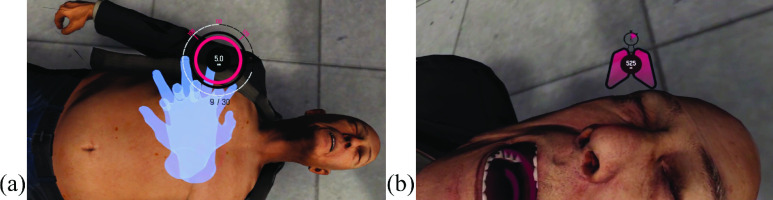
(a) Chest compression GUI (b) rescue breath GUIs (right).

GUIs implemented in the application were one of three varieties: informative, prompt, and interactive elements. The first, informative type of GUI mainly refers to the specific pieces of information that were either key facts relevant to CPR or helpful information that indicated the user’s progress through the curriculum. The second indicated that the user must perform a certain action. These were depicted using text boxes, arrows, and icons. The last type of GUI mainly consisted of two GUIs in particular – the gauges for chest compressions and rescue breaths (Fig. 4).

#### 2) Chest Compression Gauge

The chest compression gauge was developed to display the depth, rate, and number of chest compressions.

#### 3) Rescue Breath Gauge

The rescue breath gauge displayed the time left from an initial allowance of 10 seconds, the air volume, and the number of breaths performed.

### D. Usability Study

Sixteen experts with AHA BLS Instructor certification and more than 2 years of BLS teaching experience participated in the usability study. They were recruited from 6 institutions (Seoul National University Bundang Hospital, Korea University Anam Hospital, Hallym University Sacred Heart Hospital, Kyungdong University, Seongnam Citizens Medical Center, Pohang St. Mary’s Hospital), and none of them had either participated in or had any interest in the development of this device. There were no specific exclusion criteria. Participants performed a 20-minute learning mode. In the learning mode, an AI instructor appears to educate the participants. After the learning mode was completed, the training was performed in the practice mode, in accordance with real-life usage, followed the by test mode.

## III. Results

Sixteen experts participated in the usability test, of which 9 were emergency medicine specialists and 7 were emergency medical technicians. Their average total BLS education experience was 5.6 years with a standard deviation of 4.4 years. After the usability test, an expert group survey was conducted consisting of 8 items: 3 questions about the ease of use of XR-BLS, 3 questions about the delivery of training, and 2 questions about the AI instructor. The results of the survey are as follows.

Of the survey respondents, 100% answered yes (strongly agree or agree) to the questions about the ease of operating the system, whether the hands and manikins could be seen well in the VR environment, and whether the interaction with the system was clear. Concerning the delivery of CPR training, all experts answered that they could learn BLS quickly when they received BLS training using the XR-BLS simulator and that the information displayed in the XR-BLS simulator was helpful. Last, 16 (strongly agree 14, agree 2) respondents answered that the explanations and instructions of the AI instructor were easy to understand, and 14 experts answered that the interaction with the AI instructor was clear and understandable (strongly agree 10, agree 4) (Table 1).

**TABLE 1:** Results of Expert Group Survey

Whether it is easy to use	strongly agree	agree	neutral	disagree	strongly disagree	total
1. Learning to operate the system is easy	14(87.5%)	2(12.5%)	0(0%)	0(0%)	0(0%)	16(100%)
2. I was able to see my hands and manikin well in VR	8(50%)	8(50%)	0(0%)	0(0%)	0(0%)	16(100%)
3. My interaction with the system is clear and understandable	12(75%)	4(25%)	0(0%)	0(0%)	0(0%)	16(100%)
Whether training is delivered well						
1. I believe I could quickly become proficient on BLS using this system	10(62.5%)	6(37.5%)	0(0%)	0(0%)	0(0%)	16(100%)
2. The information was effective in helping me complete the tasks and scenarios	10(62.5%)	6(37.5%)	0(0%)	0(0%)	0(0%)	16(100%)
3. The instruction in the VR tutorial were organized, clear and easy to understand	14(87.5%)	1(6.3%)	1(6.3%)	0(0%)	0(0%)	16(100%)
Basic learning (Al instructor%)						
1. It was easy to understand the Al instructor’s explanations and instructions	14(87.5%)	2(12.5%)	0(0%)	0(0%)	0(0%)	16(100%)
2. My interaction with the Al instructor is clear and understandable	10(62.5%)	4(25%)	2(12.5%)	0(0%)	0(0%)	16(100%)

## IV. Discussion

With recent advances in computer technology, high-quality software functions and graphics have begun to be utilized in the education field. In particular, VR’s amplified immersion and realism are useful in this regard as it overcomes the biggest drawback of traditional education methods, which is a lack of these aspects.

Studies using VR have been conducted owing to its potential to give healthcare providers the opportunities to gain knowledge and practice techniques and procedures in a realistic environment [8], [9]. In a clinical trial for medical students, neuroanatomy was taught via both VR and conventional methods; neuroanatomy training using VR was found to potentially improve knowledge retention, increase study motivation, and reduce neurophobia [10]. With regard to procedural training, Wong *et al.* reported that post-training performance of bronchoscopic intubation, as measured by intubation time and Global rating scale, was improved in the VR group [11]. In addition, Ferlitsch *et al.* reported that virtual simulator training significantly affected technical accuracy in the early and mid-term stages of endoscopic training [12].

VR technology has also been used in CPR training. Omamah Almousa *et al.* reported that the introduction of VR into CPR training enables self-directed learning without restrictions of place, time, and number of participants [12]. A. Vankipuram *et al* [13] investigated the system usability and ease of using a VR simulator for advanced cardiac life support (ACLS) training. After conducting a questionnaire survey, both the overall system usability, which corresponds to ’whether training is delivered well’, and the overall ease of use, which refers to ’whether it is easy to use’, resulted in a mean Likert scale of approximately 3.7, which is a bit lower than that obtained in the present study, that is, 4.7 in both the categories. Although the XR-BLS simulator obtained a slightly higher score, it can be said that the results are somewhat consistent considering that ACLS training is more complex compared to BLS since the former requires a team approach.

Another study verified the usefulness of the CPR simulator using VR. Joris Nas *et al.* compared CPR quality between face-to-face CPR training and training using the VR app [5]. The VR group had a similar chest compression rate to the face-to-face method.

However, VR also has disadvantages. Since the trainee cannot directly implement the procedure on the actual subject, the ability to perform the procedure may be lower in the real world. This is supported by the fact that the group educated using VR showed inferior compression depth in the CPR study conducted by Joris Nas *et al.* [5]. Therefore, we tried to overcome the shortcomings of VR by developing an XR-BLS simulator, which includes matching real manikin to the VR environment and using it to actually perform chest compression and ventilation. An expert survey revealed the XR-BLS simulator to be convenient to use and effective in delivering information.

There are some limitations in this study. First, the expert survey results of training delivery could reflect the usefulness of the conventional BLS training program as well as the usefulness of the XR-BLS simulator itself because the conventional BLS training program has been implemented and translated into the XR-BLS simulator. Nevertheless, a convenient and easy-to-understand XR system combined with the effective BLS training program enables training without time and space constraints. In addition, AI instructors provide 1:1 training, allowing trainees to learn BLS on their own. Given these perspectives, the XR-BLS simulator is still a compelling modality for BLS training.

Second, the sample size was small and an only-8-point questionnaire was used to obtain the quantitative results, which were not presented in comparison to other devices or educational methods. This issue will be addressed in the next study, which is a multinational, pragmatic, noninferiority, randomized clinical trial (XR-BLS RCT trial) in four countries (South Korea, the United States of America, the United Kingdom, and Singapore). It has been registered with clinical trials (NCT04736888) [15].

Third, because the HMD was thick and protruded, it was difficult to practice ventilation on the manikin while wearing it. For this reason, a 3D printed mouthpiece was necessary, which created a difference when compared with the actual characteristics of a cardiac arrest patient. If a thinner and more comfortable HMD can be developed in the future, it will be used. Lastly, since no human instructors were involved in the BLS training when using the XR-BLS simulator, training may be difficult if the trainee does not rigorously follow the training steps.

## V. Conclusion and Future Work

This paper describes the design and development of an extended reality simulator for basic life support training as well as an expert usability survey. The expert survey revealed that the XR-BLS simulator was easy to use, BLS training was delivered well, the AI instructor’s explanations were useful, and the interaction between the user and the AI instructor was successful.

The next step is to compare the efficacy of the XR-based BLS training method with that of the conventional training method. A multinational RCT has been planned for this purpose, as mentioned in the previous section.

Future work on the simulator could include design improvements and additional scenario development, as well as an upgrade to the simulator to enable one-to-many training rather than one-to-one training.

In conclusion, BLS education in a more immersive and realistic environment using XR is feasible and could be useful in the context of the COVID-19 pandemic because it can be conducted without requiring instructors and trainees to assemble in a given location.

## Supplementary Materials

3D graphics, animations, and videos.

XR-BLS simulator expert usability survey.
