# Supplementary material for: Development of an Extended Reality Simulator for Basic Life Support Training
Source: IEEE J Transl Eng Health Med. 2022 Feb 16;10:4900507. doi: 10.1109/JTEHM.2022.3152365 (PMC9342859; doi:10.1109/JTEHM.2022.3152365)
Supplement: XR-BLS simulator expert usability survey. [file supp2-3152365.docx]

**XR-BLS simulator expert usability survey**

| The basic life support simulator using extended reality (XR-BLS simulator) aims to inform people of the need for CPR training and increase their confidence in performing CPR in real life.  Today's expert usability survey is the first to evaluate the usability of the currently developed XR-BLS simulator. We want to improve our services based on the information obtained from this survey. Your responses will only be used for content improvement and further research and development. Your information will not be provided to third parties. Please try a detailed answer. Thank you in advance for your valuable time. |
| --- |

| Whether it is easy to use | | Strongly Agree | Agree | Neutral | Disagree | Strongly Disagree |  |
| --- | --- | --- | --- | --- | --- | --- | --- |
| 1 | Learning to operate the system is easy | 5 | 4 | 3 | 2 | 1 |  |
| 2 | I was able to see my hands and manikin well in VR. | 5 | 4 | 3 | 2 | 1 |  |
| 3 | My interaction with the system is clear and understandable | 5 | 4 | 3 | 2 | 1 |  |
| Whether training is delivered well | | Strongly Agree | Agree | Neutral | Disagree | Strongly Disagree |  |
|  |  |  |  |  |  |  |  |
| 1 | I believe I could quickly become proficient on BLS using this system | 5 | 4 | 3 | 2 | 1 |  |
| 2 | The information was effective in helping me complete the tasks and scenarios | 5 | 4 | 3 | 2 | 1 |  |
| 3 | The instructions in the VR tutorial were organized, clear and easy to understand. | 5 | 4 | 3 | 2 | 1 |  |

| Basic Learning (AI Instructor) | | Strongly Agree | Agree | Neutral | Disagree | Strongly Disagree |
| --- | --- | --- | --- | --- | --- | --- |
| 1 | It was easy to understand the AI instructor's explanations & instructions. | 5 | 4 | 3 | 2 | 1 |
| 2 | My interaction with the AI instructor is clear and understandable | 5 | 4 | 3 | 2 | 1 |
